# Glycosylation of oral bacteria in modulating adhesion and biofilm formation

**DOI:** 10.1080/20002297.2025.2486650

**Published:** 2025-04-08

**Authors:** Simeng Yi, Yingyu Liu, Qinrui Wu, Danning Zhao, Zhengyi Li, Xian Peng, Ga Liao, Shida Wang

**Affiliations:** a State Key Laboratory of Oral Diseases and National Center for Stomatology & National Clinical Research Center for Oral Diseases, West China Hospital of Stomatology, Sichuan University, Chengdu, China; b Department of Cariology and Endodontics, West China Hospital of Stomatology, Sichuan University, Chengdu, China; c State Key Laboratory of Oral Diseases & National Center for Stomatology & National Clinical Research Center for Oral Diseases & Department of Information Management & Department of Stomatology Informatics, West China Hospital of Stomatology, Sichuan University, Chengdu, China; d Department of General Dentistry, West China Hospital of Stomatology, Sichuan University, Chengdu, China

**Keywords:** Oral bacteria, glycosylation, glycosyltransferase, adhesion, biofilm

## Abstract

**Background:**

Glycosylation is a ubiquitous biochemical process that covalently attaches glycans to proteins or lipids, which plays a pivotal role in modulating the structure and function of these biomolecules.  This post-translational modification is prevalent in living organisms and intricately regulates various biological processes, including signaling transduction, recognition, and immune responses.  In the oral environment, bacteria ingeniously use glycosylation to enhance their adhesion to oral surfaces, which is a key step in biofilm formation and subsequent development.  This adhesion process is intimately associated with the onset and progression of oral diseases, including dental caries and periodontal disease.

**Objective:**

This review aims to describe the types and mechanisms of glycosylation in oral bacteria, and to understand the role of glycosylation in the adhesion, biofilm formation and virulence of oral bacteria.

**Methods:**

We reviewed articles on glycosylation in a variety of oral bacteria.

**Conclusion:**

In cariogenic bacteria and periodontopathic pathogens, glycosylation facilitates adhesion and subsequent biofilm maturation on tooth surface.   Distinct glycosylation patterns in oral bacteria shape biofilm structure and function, influencing microbial interactions and community stability.   Pathogen-specific glycosylation signatures enhance virulence and ecological competitiveness, contributing to disease progression. Glycosylation plays a critical role in bacterial virulence and community  interactions, with significant implications for oral health and disease development.

## Introduction

The oral microenvironment is a remarkably complex and dynamic ecosystem, harboring a diverse array of microorganisms crucial for maintaining oral health and contributing to oral diseases. This intricate microbial community, known as the oral microbiome, comprises over 700 bacterial species, along with various fungi, viruses, and archaea [1]. Among these microbes, bacteria are the most abundant and well-characterized, with their intricate interactions and metabolic activities shaping the ecological balance of the oral cavity. There is growing evidence that *Streptococcus mutans* [2], *Porphyromonas gingivalis* [3], and *Fusobacterium nucleatum* [4] are associated with a variety of oral diseases such as dental caries and periodontitis. Central to the pathogenesis of these oral diseases is the ability of bacteria to adhere to and colonize the teeth, gum tissue, dental implants, and other structures within the oral cavity [5,6]. Oral bacteria ingeniously adhere to dental surfaces through various mechanisms, gradually forming intricate biofilm communities [7,8]. The composition and architecture of these biofilms are highly complex, playing a vital role in bacterial growth [9], environmental adaptation [10], and resistance to external stresses [11].

Glycosylation, defined as the covalent attachment of carbohydrates to proteins or lipids, is a pervasive post-translational modification in oral bacteria [12–15]. This process is catalyzed by specific enzymes called glycosyltransferases, which transfer sugar residues from activated donor molecules to specific amino acid residues (in the case of protein glycosylation) or lipid molecules (in the case of lipid glycosylation) [12,15–17]. The resulting glycoconjugates, such as glycoproteins and glycolipids, exhibit altered structural and functional properties, contributing to various biological processes, including cell-cell recognition, adhesion, signaling, and immune modulation [18–23]. Oral bacteria possess a diverse array of glycosylation systems, enabling them to modify their surface proteins and lipids with an assortment of carbohydrate structures [16] (Table 1). These glycoconjugates serve as adhesins, facilitating the attachment of bacteria to specific receptors on host cells, other microbial surfaces, and non-living substrates such as salivary proteins, exposed dentin, and root connective tissue [24,25]. Glycosylation of surface molecules in oral bacteria has emerged as a key determinant in mediating their adhesion to tooth surfaces, oral mucosa, and other microbial surfaces. Moreover, this process actively participates in biofilm development and maintains the delicate ecological balance of the oral microbiota. Biofilm refers to bacterial communities living in extracellular polymeric substances (EPS) secreted by themselves. The EPS consists mainly of polysaccharides, proteins, lipids, and nucleic acids (eDNA), which help to form the overall scaffolding of the biofilm [26,27]. Biofilm formation is a complex multi-stage process that starts with the initial adhesion of bacterial cells to the surface, followed by cell proliferation, formation of exopolysaccharides and maturation of the biofilm into a highly organised community [27,28]. There are complex interactions among oral bacteria, which predominantly occupy their respective ecological niches in a dynamic equilibrium. However, as the microbial community develops, by-products of microbial metabolism and host immune responses can alter the local environment, potentially leading to dysbiosis [29]. In most cases, biofilms provide a protective environment for bacterial growth, enabling them to withstand mechanical forces, host immune responses, and antimicrobial agents [30–33]. The glycosylation of bacterial surface molecules is of critical importance at each stage of biofilm formation, from the initial attachment phase to the establishment of a stable biofilm architecture [34–36]. Many studies have underscored the significance of glycosylation in the adhesion and biofilm formation of oral bacteria. Among them, the oral pathogens *Streptococcus mutans* and *Porphyromonas gingivalis* utilize glycosylation to enhance their virulence. *Streptococcus mutans*, a major contributor to dental caries, modifies a variety of substrate proteins such as Cnm and WapA via the Pgf glycosylation system. *Porphyromonas gingivalis*, a key pathogen in periodontal disease, employs glycosylated surface proteins, including fimbriae and gingipains, to facilitate adhesion to and invasion of host cells.

**Table 1.** Glycosylation substrates of oral bacteria.

Organism	Glycosylation substrates	Glycosyltransferase	Function of proteins	Reference	
*Streptococcus mutans*	Cnm	PgfS, PgfE, PgfM1 and PgfM2	Bacterial adhesion		Avilés-Reyes et al. [43], Avilés-Reyes et al. [44]
*Streptococcus mutans*	WapA	PgfS, PgfE, PgfM1 and PgfM2	Aggregation; biofilm formation		Zhu et al. [23], Li et al. [45]
*Streptococcus mutans*	SpaP	PgfS, PgfE, PgfM1 and PgfM2	Biofilm formation; bacterial adhesion		Kelly et al. [46]
*Porphyromonas gingivalis*	Arg-gingipain proteases (Rgp) and Lys-gingipain proteases (Kgp)	VimF	Pathogenicity		Vanterpool et al. [47], Nakayama [48]
*Porphyromonas gingivalis*	Hemoglobin-binding protein 35	Unknown	Cell surface attachment		Shoji et al. [49]
*Streptococcus parasanguinis*	Fap1	SecY2, Orf1, Orf2, Orf3, SecA2, Gtf1, Gtf2	Biofilm formation; bacterial adhesion		Peng et al. [19], Wu et al. [50]
*Fusobacterium nucleatum*	Fap2	Unknown	Co-aggregation, cell adhesion and pathogenicity		Coppenhagen-Glazer et al. [51]
*Tannerella forsythia*	TfsA and TfsB	GtfS, GtfM, GtfI, GtfL, GtfE	Pathogenicity		Sakakibara et al. [52]
*Actinomyces oris*	GspA	LytR-CpsA-Psr (LCP)	Biofilm formation under cation stress		Siegel et al.[53], Wu et al. [54]
*Staphylococcus aureus*	Lipoteichoic acid (LTA) anchor	YpfP	Biofilm formation; penetration of the blood-brain barrier		Fedtke et al. [55], Sheen et al. [56]
*Staphylococcus aureus*	Wall teichoic-acid (WTA)	TarM	Nasal colonization		Winstel et al.[57], Xia et al. [58]

The study of glycosylation in oral bacteria has gained momentum in recent years, driven by advances in analytical techniques and a growing appreciation of its biological significance. Mass spectrometry-based approaches, such as liquid chromatography-tandem mass spectrometry (LC-MS/MS), have enabled the identification and characterization of novel glycoproteins and glycolipids in oral bacteria [37,38]. Additionally, genetic and biochemical studies have provided insights into the glycosylation pathways and enzymes involved in the synthesis and attachment of glycans to bacterial surface molecules [15,39–41]. These insights have not only enhanced our comprehension of the molecular mechanisms of bacterial adhesion and biofilm formation, but have also facilitated the development of novel targeted therapeutic strategies. Despite the progress made in this field, many aspects of glycosylation in oral bacteria remain poorly understood. The functional significance of specific glycan structures, the regulation of glycosylation pathways in response to environmental cues, and the interplay between glycosylation and other virulence factors are among the key questions that warrant further investigation. Moreover, the role of glycosylation in shaping the interactions between different bacterial species within the oral microbiome and its impact on the host immune response are areas of active research [42].

This review intends to offer a comprehensive overview of the current understanding of the role of glycosylation in regulating the adhesion and biofilm formation of oral bacteria. We discuss the types of glycosylation found in bacteria, with a focus on protein and lipid glycosylation, and their functional implications. We then delve into the specific glycosylation systems and glycoconjugates identified in key oral bacterial species and their contributions to adhesion and biofilm formation. The potential therapeutic and diagnostic applications of targeting bacterial glycosylation will also be discussed. Finally, we highlight the emerging trends and future directions in this field, emphasizing the need for interdisciplinary approaches to unravel the complexity of glycosylation in the oral microbiome. Elucidating the glycosylation sites in oral bacteria is expected to pave the way for targeted interventions to attenuate the virulence or adhesion capacity of oral pathogens, opening up new avenues for maintaining oral health and treating oral diseases.

## Glycosylation in bacteria

### Glycosylation of proteins

**Figure 1.**
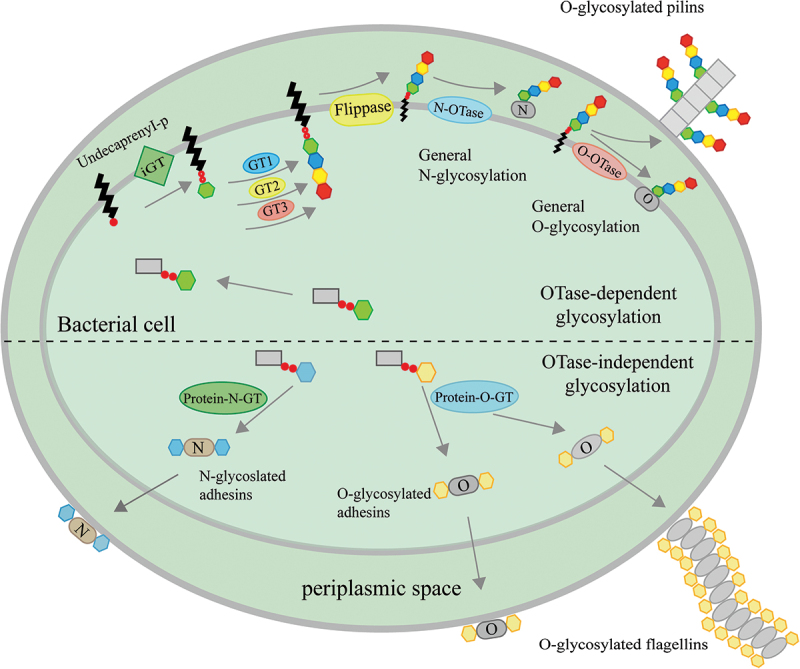
The general landscape of bacterial protein glycosylation.

Protein glycosylation is a common post-translational modification that plays a pivotal role for life activities in prokaryotes and eukaryotes. Protein glycosylation in bacteria has been shown to be important for physiological functions including cell recognition, adhesion and interaction [59–62]. There are two main types of protein glycosylation modification: N-glycosylation and O-glycosylation (Figure 1), in which the sugar molecule is attached directly to the amino terminus of an asparagine (N-glycosylation) or to the hydroxyl group of a serine or threonine (O-glycosylation). Bacterial N-glycosylation, originally discovered in *Campylobacter jejuni*, is dependent on the pgl glycosylation pathway [63]. The Pgl operon contains 12 open reading frames (ORFs) encoding several glycosyltransferases and glycobiosynthetic enzymes [64]. These ORFs generate a lipid-linked heptasaccharide precursor, and PglB is responsible for the transfer of the heptasaccharide to the target asparagine [65]. Mutations in the *pgl* locus (especially *pglB*) affect protein glycosylation in *Campylobacter jejuni*, reducing bacterial adhesion and invasiveness and thus greatly reducing bacterial colonisation [66,67]. *Haemophilus influenzae* produces two important high-molecular-weight (HMW) adhesins, HMW1 and HMW2, which mediate its attachment to human epithelial cells [68,69]. Glycosylation of HMW1/2 is dependent on HMW1/2C, and HMW1C has been shown to possess glycosyltransferase activity that catalyses the N-linked glucosylation or galactosylation of asparagine in HMW1, which is essential for HMW1 stability and attachment ability [70]. Bacterial O-glycosylation is poorly characterized because of the heterogeneity of O-glycosyltransferases and O-oligosyltransferases and the complexity of the stepwise attachment of glycoconjugates of the system [71,72].

Bacteria exhibit diverse glycosylation patterns, which can be broadly categorized into two main pathways based on the assembly and transfer of glycans to the acceptor protein: the oligosaccharyltransferase (OTase)-dependent and the OTase-independent pathways [73]. OTases are glycosyltransferases (GTases) that catalyze the transfer of an oligosaccharide from a lipid donor to an acceptor molecule [73]. The OTase-dependent glycosylation mechanism involves the transfer of monosaccharides to the lipid carrier undecaprenyl phosphate (Und-P) by the initiating glycosyltransferase (iGT). GTase in the cytoplasm transfers the monosaccharides to the lipid carrier in a successive manner, forming a lipo-conjugated carbohydrate chain. This chain is then flipped to the outside of the cell membranes by flippase, and finally attached to specific amino acid residues by OTase [73]. PglB, a major OTase involved in N-linked glycosylation, has been identified in several bacteria, including *Campylobacter jejuni*, *Wolinella succinogenes*, and *Desulfovibrio desulfuricans* [74,75]. PglL, an OTase responsible for O-linked glycosylation, is commonly found in various bacterial species such as *Neisseria meningitidis*, *Pseudomonas aeruginosa*, and *Neisseria gonorrhoeae* [75,76]. PglL is also capable of catalyzing the O-linked glycosylation of pilin proteins [76]. This pathway is similar to the process of N-glycosylation that occurs in bacteria such as *Campylobacter jejuni*, and to the synthesis of lipopolysaccharide (LPS) [77–79]. In the OTase-independent glycosylation pathway, monosaccharides are sequentially transferred onto the target protein one at a time through the consecutive action of *N*- and O-glycosyltransferases (PGTases) [73]. This pathway is commonly employed in glycosylation of flagellar structural protein and adhesins [80–82]. For example, *Helicobacter pylori* flagella, consisting of FlaA and FlaB, is post-translationally modified by glycosylation with pseudaminic acid (Pse) [83]. The elevated level of O-glycosylation in FlaA was due to elevated levels of Pse in *Helicobacter pylori* 0518 mutant, which affects pathogen motility and bacterial colonisation efficiency [84]. The decrease in the glycosylation level of *Pseudomonas syringae* flagella affects bacterial invasiveness of the soybean host [85]. The flagellar glycosylation system was found in many Gram-negative bacteria, but in Gram-positive bacteria only in the genera *Clostridium* and *Listeria* [86]. The flagellin protein of *Listeria monocytogenes* is posttranslationally modified with O-linked N-acetylglucosamine (GlcNAc) at up to six sites [86]. In *Clostridium difficile*, the glycan was attached to the flagellin protein backbone in O linkage via a HexNAc residue [87].

Bacterial nucleotide-activating sugars provide nutrients for the protein glycosylation pathway. In OTase-dependent protein glycosylation, an initiating GTase first attaches a monosaccharide to an Und-P molecule on the cytoplasmic face of the inner membrane. The rest of the oligosaccharide is then synthesized by the sequential action of cytoplasmic GTases (GT1, GT2, and GT3). Afterward, a flippase transports the lipid-linked oligosaccharide from the cytoplasmic face to the periplasmic face of the inner membrane. The oligosaccharide is attached to an asparagine residue by an N-OTase, and to a serine/threonine residue by an O-OTase. In OTase-independent glycosylation, Protein N-GTases and Protein O-GTases, which are cytoplasmic GTases, transfer mono- or di-hexoses onto target proteins. Protein N-GTases primarily modify adhesins, while Protein O-GTases modify both adhesins and flagellins. Gram-positive bacteria also utilize a similar pathway for glycosylation, and for simplicity, only Gram-negative bacteria are described.

### Glycosylation of lipids

Lipid glycosylation is commonly used to describe the process by which carbohydrate molecules are covalently linked to lipid molecules by glycosyltransferases [88], resulting in the formation of glycolipids. Glycolipids contain one or more monosaccharide residues that are linked by glycosidic bonds with a water-repellent portion, which may be one of the following: acylglycerol, sphingosine (a long-chain aliphatic amino alcohol), ceramide (N-acylsphingosine), or isoprenyl phosphate [89]. In nature, glycolipids can be broadly classified into two major categories based on the hydroxyl groups in their components: glyceroglycolipids and glycosphingolipids (GSLs) [13,90]. Glyceroglycolipids have a glycosylacylglycerol structure similar to that of phospholipids, with the main chain being glycerol, containing fatty acids but not phosphorus and compounds such as choline [90]. Sugar residues are attached to the C-3 position of 1,2-glycerol diesters through glycosidic bonds to form glycosylglyceride molecules. Glyceroglycolipids are found in the nervous tissue of animals, plants and microorganisms. They are the primary glycolipids in plants [91] and are a common component of bacterial membranes, particularly in gram-positive bacteria [92,93]. Sphingolipid molecules consist of three basic structural components: sphingomyelin, a long-chain glycol with an amino group of around 18 C; a long-chain fatty acid, ranging from approximately 18 to 26 C, which forms an amide bond with sphingomyelin and is termed ceramide; and a polar group head, usually linked to the hydroxyl group of the first carbon atom of sphingomyelin [90,94]. Different types of sphingolipids are formed due to different polar groups, e.g. those containing phosphoric acid are called sphingomyelin, and those containing sugar groups are called glycosphingolipid [90]. Glycosphingolipids are involved in intercellular communication and some of them function as receptors for viruses and bacterial toxins [95,96].

Glycolipids are important in maintaining the structural integrity of bacteria and are also associated with bacterial pathogenicity. Lipoteichoic acid (LTA) is a glycolipid-anchored phosphopolyol polymer found in Gram-positive bacteria [97]. It consists of a hydrophilic portion made up of polyglycerolphosphate chains and a hydrophobic portion that contains a glycolipid anchor (e.g. diacylglycerol). The glycolipid anchor serves to attach LTA to the cell membrane, playing a crucial role in maintaining the stability and integrity of the cell membrane [92]. Glycosyltransferase YpfP in *Staphylococcus aureus* transfers two glucose units from UDP-Glc to diacylglycerol (DAG), forming glucosyl (β1–6) glucosyl (β1–3) DAG (Glc2-DAG) [98], which subsequently forms the LTA anchor. Mutations in *ypfP* lead to reduced LTA synthesis, impaired biofilm formation [56] and reduced ability to cross the blood-brain barrier [57]. Lipopolysaccharide (LPS) is a highly acylated glycolipid on the outer membrane of Gram-negative bacteria, which consists of lipid A, a core oligosaccharide and an O-antigen polysaccharide [99]. LPS containing a certain amount of O-antigen is called Smooth-LPS and LPS lacking O-antigen is called Rough-LPS [100]. The process of LPS synthesis in the cytoplasm and its transport has been extensively studied in *Escherichia coli*. At the inner membrane of the cell membrane, KdtA attaches 3-deoxy-D-manno-octulosonic acid (Kdo) to the reducing end phosphate group of lipid A to form the Kdo-lipid A complex [101]. Subsequently, other parts of the core oligosaccharide are gradually added to Kdo in the periplasmic space, resulting in the formation of the lipid A-core oligosaccharide complex [102]. LPS is widely distributed on the cell surface, forming a protective barrier that allows selective permeability and shields the cell from harmful substances such as antibiotics and bile salts [103].

## The role of glycosylation in oral bacteria

**Figure 2.**
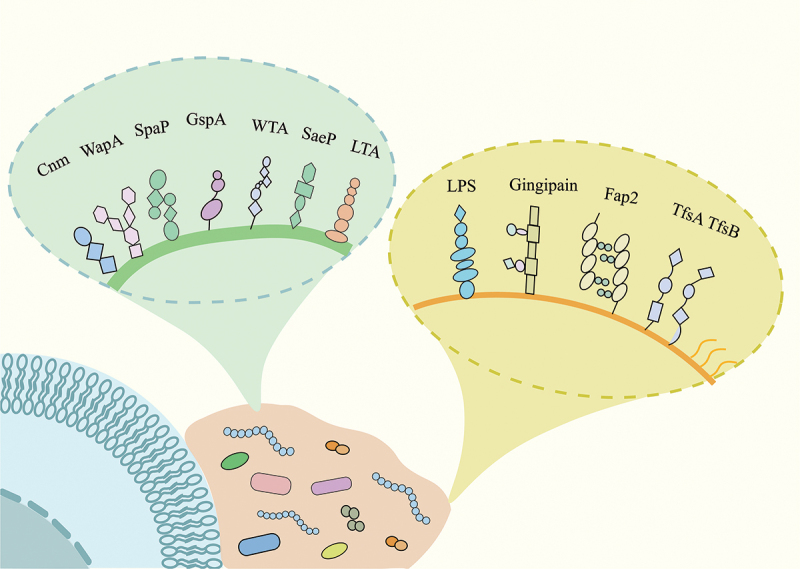
Glycosylated molecules during oral bacterial adhesion.

Oral bacteria have evolved sophisticated mechanisms to adhere to various surfaces within the oral cavity, forming complex biofilm communities. Glycosylation of bacterial surface molecules is a crucial factor in mediating adhesion and biofilm formation. Cariogenic pathogens, such as *Streptococcus mutans*, and periodontal pathogens, including *Porphyromonas gingivalis* and *Fusobacterium nucleatum*, employ a wide range of glycosylated molecules to facilitate their attachment to host tissues and other microbes (Figure 2). These glycoconjugates, which include surface proteins, lipoteichoic acids, and lipopolysaccharides, play essential roles in bacterial virulence and the pathogenesis of oral diseases.

Given the significant role that oral bacteria play in the development of oral diseases, this review focuses on the glycosylation mechanisms in these microorganisms. Understanding these processes is crucial not only for comprehending their pathogenesis but also for developing targeted strategies for prevention and treatment of oral diseases.

### Glycosylation in cariogenic bacteria

A variety of caries-causing pathogens exist in the oral cavity, such as *Streptococcus mutans*, *Streptococcus sobrinus* and *Lactobacillus* [104,105]. Of these, *Streptococcus mutans* has been the most extensively studied.

*Streptococcus mutans* uses various glycosylated surface molecules to facilitate its adhesion to the tooth surface and the formation of biofilms. The Pgf glycosylation mechanism is currently well studied in *Streptococcus mutans*. The pgf system possesses a broad range of modification capabilities and may mediate post-translational modifications of both core and non-core proteins of *Streptococcus mutans* by glycosylation, which is critical for biofilm formation, protein stability, cell invasiveness and virulence [106]. Pgf glycosylation machinery consists of four enzymes: PgfS, PgfM1, PgfE, and PgfM2 [107]. Although experimental characterization is lacking, bioinformatics analysis predicts that PgfM1 and PgfM2 are membrane-embedded enzymes, PgfS is a GT-A type glycosyltransferase, and PgfE is a GlcNAc-4-epimerase [107]. At least four targets of the pgf system have been identified: WapA, SpaP, Cnm, and GtfC. Notably, all four proteins have been implicated in the initial steps of *Streptococcus mutans* colonisation in the oral cavity. WapA and SpaP are core collagen-binding proteins (CBP) of *Streptococcus mutans* [108,109], and Cnm is a non-core CBP present in approximately 20% of clinical isolates [110,111]. WapA has been reported to affect cell-cell aggregation and biofilm structure in pathogenic bacteria [23,45]. SpaP, also known as P1, antigen I/II, is a conserved adhesion protein [112]. It promotes adhesion of *Streptococcus mutans* to saliva-coated tooth surfaces by specifically interacting with salivary agglutinin [46]. Deleting *spaP* gene in *Streptococcus mutans* reduced virulence in a rat caries model [113] as well as alterations in its cell surface properties [114] and biofilm-formation characteristics [115]. Cnm is a protein located on the cell surface, comprising an N-terminal secretion signal, a conserved collagen-binding, a threonine-rich domain, and a C-terminal LPXTG motif for cell wall anchoring [116]. Cnm is a virulence factor associated with bacterial invasion of oral epithelial cells and colonisation of heart valves [117,118].

### Glycosylation in periodontal pathogens

Periodontal pathogens, such as *Porphyromonas gingivalis*, *Fusobacterium nucleatum* and *Tannerella forsythia*, employ various glycosylated surface molecules attach to host tissues and form biofilms [51,119,120]. *Porphyromonas gingivalis* is the most studied of all periodontal pathogens. *Porphyromonas gingivalis* lipopolysaccharides (LPS) contain both O-side chain lipopolysaccharides (O-LPS) and anionic lipopolysaccharides (A-LPS) [121]. Glycosyltransferases, which are closely related to lipopolysaccharide synthesis, include the *gtfB* gene that is homologous to glycosyltransferase 1 in several bacteria [121]. The *gtfB* mutant strain, deficient in O-LPS and A-LPS, exhibits phenotypic defects including the loss of surface-associated gingipain, enhanced self-aggregation, and increased biofilm formation, indicating that LPS polysaccharides are essential for gingipain attachment, modulation of self-aggregation, and biofilm formation [120]. Gingipain is a cysteine protease and a major virulence factor of *Porphyromonas gingivalis* [122]. The gingipain family consists of three proteases that exhibit strict specificity for arginine or lysine substrates [123]. RgpA and RgpB (arginine-specific gingipains) cleave only at Arg-Xaa peptide bonds, while Kgp (lysine-specific gingipain) exclusively hydrolyzes Lys-Xaa peptide bonds [124]. Furthermore, glycosylation regulates both the activity and attachment of gingipain, the major virulence factor of *Porphyromonas gingivalis* [125]. The VimF glycoprotein is a galactosyltransferase [126]. Gingipain with low activity was detected on membranes of *vimF* mutant strains, whereas deletion of the *gtfB* gene resulted in complete loss of surface-associated gingipain [124,127], suggesting that *VimF* and *gtfB* may use different mechanisms to alter gingipain. *VimF* gene deletion strains showed reduced Arg-X- and Lys-X-specific proteolytic activities compared to the wild-type *Porphyromonas gingivalis* W83 strain [127]. Although the gene expression of the arginine- and lysine-dependent gingipains in this mutant remained unaffected [127], altered glycosylation resulted in a reduction of their activity [127].

In addition, *Fusobacterium nucleatum* and *Tannerella forsythia* surface glycoproteins affected aggregation and adhesion. The cell surface glycoprotein Fap2 of *Fusobacterium nucleatum* is a galactose-inhibitable adhesion protein involved in its co-aggregation and cell adhesion [52]. Fap2 mutants fail to show galactose-inhibited co-aggregation with *Porphyromonas gingivalis* and are defective in binding to Human embryonic kidney (HEK) 293T cells [52]. The S-layer of *Tannerella forsythia*, which consists of two glycoproteins, TfsA and TfsB, is highly glycosylated with a unique, complex decasaccharide [120]. S-layer glycoproteins are bacterial virulence factors. The S-layer has been shown to mediate hemagglutination [128], as well as adhesion and invasion of human gingival epithelial cells [53].

### Glycosylation in other oral bacteria

Many commensal bacteria, including S*treptococcus parasanguinis, Streptococcus gordonii, Actinomyces oris* and *Staphylococcus aureus*, colonize the oral cavity and utilize glycosylated surface molecules to facilitate the initiation and progression of disease. Fap1-like serine-rich glycoproteins are conserved in *Streptococci* and *Staphylococci*, and play essential roles in bacterial biofilm formation and pathogenesis [34]. Fap1 (fimbriae-associated protein 1) repeat sequence is modified by O-linked glycans in *Streptococcus parasanguinis* [129]. Fap1 consists of a cluster of seven genes encoding for glycosyltransferases (Gtf1, Gtf2) and secreted proteins (SecA2, SecY2, Gap1–3). These enzymes are involved to varying degrees Fap1 glycosylation and secretion, with Gap3 playing a particularly crucial role in fimbrial assembly, bacterial adhesion and biofilm formation [19]. *Streptococcus gordonii* adhesin GspB mediates adhesion between bacteria and extracellular matrix, and bacteria and host cells. It was found that the protein is O-glycosylated by GtfA/B, Nss and Gly glycosyltransferases and is subsequently secreted into the extracellular domain with the assistance of three helper Sec proteins (Asp1–3) [130–132]. Deletion of any of the enzymes GtfA/B, Nss and Gly affects the modification of GspB and reduces bacterial adhesion [133–135]. Gram-positive pathogens, including *Actinomyces oris*, employ the conserved cysteine transpeptidase Sortase to catalyze the covalent attachment of numerous glycosylated adhesins to the cell wall. Wu et al. proposed a model for GspA glycosylation involving the coordinated activity of LytR-CpsA-Psr (LCP) and SrtA. In this model, LCP, functioning as a glycosyltransferase, mediates the ligation of specific sugar chains to GspA, while SrtA catalyzes the subsequent attachment of glycosylated GspA to bacterial peptidoglycan. Notably, when SrtA expression is downregulated or its activity is inhibited, GspA accumulates and undergoes over-glycosylation within the membrane, ultimately leading to cell death [54,55]. *Staphylococcus aureus* uses a membrane-associated three-component glycosylation system for LTA glycosylation, involving CsbB (Und-GlcNAc charging enzyme), GtcA (flippase for substrate transport), and YfhO (LTA-specific glycosyltransferase adding α-GlcNAc moieties) [136]. The deletion of *ypfP*, a gene essential for glycolipid biosynthesis in LTA, reduced LTA content by 87% in *Staphylococcus aureus* SA113 and inhibited its biofilm formation on hydrophobic surfaces due to impaired adhesion [56]. LTA supports matrix and biofilm maturation, while eDNA cooperates with EPS in early biofilm development [137]. The results of Mlynek et al. showed that eDNA and polysaccharide intercellular adhesins act synergistically in *Staphylococcus aureus* biofilm formation and bacterial aggregation [138]. The SaeP protein of *Staphylococcus aureus*, a membrane-attached lipoprotein, interacts with eDNA, reinforcing the biofilm electrostatic net [139].

These studies reveal the key role of oral bacteria in adhesion and biofilm formation, especially *Streptococcus* and *Staphylococcus*. Through glycosylation modifications, these bacteria are able to modulate the affinity of their surface molecules, thus contributing to their settlement and pathogenicity in the oral environment.

In the diagram, green represents Gram-negative bacteria, while yellow denotes Gram-positive bacteria. Bacteria, along with secreted polysaccharides and other substances, collectively form a biofilm structure (orange) that adheres to the host cell surface (blue). A variety of glycosylated molecules play a crucial role in this adhesion process. Examples include Cnm, WapA, and SpaP from *Streptococcus mutans*; GspA from *Actinomyces oris*; WTA, SaeP, and LTA from *Staphylococcus aureus*; LPS and Gingipain from *Porphyromonas gingivalis*; Fap2 from *Fusobacterium nucleatum*; and TfsA and TfsB from *Tannerella forsythia*.

## Conclusions and perspectives

Glycosylation is a fundamental regulatory mechanism governing the ecological dynamics of oral microbiota. Through enzymatic modification of surface proteins and lipids, this post-translational process plays a dual role in maintaining oral health and driving disease progression. In cariogenic bacteria and periodontopathic pathogens, glycosylation facilitates adhesion and subsequent biofilm maturation on tooth surface. Distinct glycosylation patterns in oral bacteria shape biofilm structure and function, influencing microbial interactions and community stability. Pathogen-specific glycosylation signatures enhance virulence and ecological competitiveness, contributing to disease progression. Overall, glycosylation plays a critical role in bacterial virulence and community interactions, with significant implications for oral health and disease development.

Research into glycosylation regulation in oral bacteria may lead to novel strategies for maintaining oral health, including interventions targeting glycosylation pathways to reduce biofilm formation and bacterial virulence. Further exploration of the molecular mechanisms involved, along with the use of advanced technologies like glycomics and proteomics, will be key to understanding glycosylation’s role in oral diseases and developing more effective treatments.
